# Structural Characterisation and Assessment of the Novel *Bacillus amyloliquefaciens* RK3 Exopolysaccharide on the Improvement of Cognitive Function in Alzheimer’s Disease Mice

**DOI:** 10.3390/polym13172842

**Published:** 2021-08-24

**Authors:** Ravi Gangalla, Sampath Gattu, Sivasankar Palaniappan, Maqusood Ahamed, Baswaraju Macha, Raja Komuraiah Thampu, Antonella Fais, Alberto Cincotti, Gianluca Gatto, Murali Dama, Amit Kumar

**Affiliations:** 1 Department of Microbiology, Kakatiya University, Warangal 506009, India; g.ravi320@gmail.com; 2 Department of Zoology, School of Life Sciences, Periyar University, Salem 636011, India; sampath.gattu2@gmail.com; 3 Department of Environmental Science, School of Life Sciences, Periyar University, Salem 636011, India; 4 Department of Physics and Astronomy, College of Science, King Saud University, Riyadh 11451, Saudi Arabia; mahamed@ksu.edu.sa; 5 Medicinal Chemistry Division, University College of Pharmaceutical Sciences, Kakatiya University, Warangal 506009, India; baswarajpharma1@gmail.com; 6 Department of Life and Environmental Sciences, University of Cagliari, Monserrato, 09042 Cagliari, Italy; fais@unica.it; 7 Department of Mechanical, Chemical and Material Engineering, University of Cagliari, Via Marengo 2, 09123 Cagliari, Italy; alberto.cincotti@dimcm.unica.it; 8 Department of Electrical and Electronic Engineering, University of Cagliari, Via Marengo 2, 09123 Cagliari, Italy; gatto@unica.it (G.G.); amit.kumar@unica.it (A.K.); 9 Institute for Plant Cell Biology and Biotechnology, Heinrich Heine University Düsseldorf, 40225 Düsseldorf, Germany; murali.dama@gmail.com

**Keywords:** Alzheimer’s disease, cognitive function, exopolysaccharide, *Bacillus*, biopolymer, biomedical application

## Abstract

In this study *Bacillus amyloliquefaciens* RK3 was isolated from a sugar mill effluent-contaminated soil and utilised to generate a potential polysaccharide with anti-Alzheimer’s activity. Traditional and molecular methods were used to validate the strain. The polysaccharide produced by *B. amyloliquefaciens* RK3 was purified, and the yield was estimated to be 10.35 gL^−1^. Following purification, the polysaccharide was structurally and chemically analysed. The structural analysis revealed the polysaccharide consists of α-d-mannopyranose (α-d-Man*p*) and β-d-galactopyranose (β-d-Gal*p*) monosaccharide units connected through glycosidic linkages (i.e., β-d-Galp(1→6)β-d-Galp (1→6)β-d-Galp(1→2)β-d-Galp(1→2)[β-d-Galp(1→6)]β-d-Galp(1→2)α-d-Manp(1→6)α-d-Manp (1→6)α-d-Manp(1→6)α-d-Manp(1→6)α-d-Manp). The scanning electron microscopy and energy-dispersive X-ray spectroscopy imaging of polysaccharides emphasise their compactness and branching in the usual tubular heteropolysaccharide structure. The purified exopolysaccharide significantly impacted the plaques formed by the amyloid proteins during Alzheimer’s disease. Further, the results also highlighted the potential applicability of exopolysaccharide in various industrial and pharmaceutical applications.

## 1. Introduction

Microorganisms produce exopolysaccharides (EPS) by utilising different nutrient sources. EPS are ubiquitous and have been reported from diverse sources of microorganisms [1]. The different EPS are functionally characterised as significant polymeric substances, which are extracellularly produced by various microbial species [2]. EPS can execute different essential biological functions in various organisms. Microbial EPSs are also known to exhibit antioxidant properties, anti-ulcer and anti-toxin activity [3]. They are classified into homopolysaccharides with only one or the same type of sugar molecules, e.g., cellulose, alternan, pullulan, mutant, levan, dextran and curdlan. Others are called heteropolysaccharides, with two or more/different sugar moieties attached, e.g., gellan and xanthan [4,5]. Similarly, the structure of the microbial EPS is also mainly composed of monomer units linked by glycosidic linkages [6].

Different microorganisms produce a differential amount of exopolysaccharide content according to their surroundings and metabolism [7]. Some *Bacillus* sp. are known to produce significantly more quantities of EPS than *Lactobacillus* sp. and also the *Bacillus* sp. can produce more than one type of EPS [8]. Nature has various EPS-producing microbes such as *B. licheniformis*, *B. amyloliquefaciens*, *A. infernus* and some *Luconostoc* sp. [9]. EPS is purely organic, possesses higher stability to extreme conditions and is environmentally friendly and also biodegradable [10]. Novel exopolysaccharides are extremely attractive, and it has been shown that microbial EPS have a wide range of applications in various fields containing textile, oil recovery, food, pharmaceutical, tissue engineering, cosmetics and chemical industries [11]. In addition, microbial EPS possesses great applications in the therapeutic industries [12]. The substantial immunomodulatory and anticancer characteristics of EPS have led the path for its use in a variety of biological and therapeutic applications. 

In addition to controlled drug administration in EPS, potential applications include vaccines, adjuvants and diagnostic imaging systems [13]. In commercial pharmaceuticals and medical devices, many potential applications are expected to be developed. Based on past and current results relevant to the medical and pharmaceutical fields, the state of EPS in medical applications is very diverse [14]. Due to their specific material qualities, bacterial polysaccharides are effective biomaterials. EPS has several advantages, including lubricity, rheological and viscoelasticity, cationic interaction, ionic strength, crosslinking, gelling, water retention and stability under a variety of circumstances [15,16]. For instance, the critical ability of microbial cellulose in medical applications can be moulded into various forms without losing its beneficial properties. By forming into long hollow tubes, these tubes can be used as replacement structures in various areas like the cardiovascular system, digestive tract, urinary tract and windpipe [17]. Microbial cellulose can also be used for internal treatments such as bone grafting and other tissue techniques and regeneration [18].

On the other hand, alginate extracted from bacteria has its own physical and chemical heterogeneity, which affects its quality and generates different applications [19]. Alginates with all outstanding properties have been investigated for biomedical applications. Alginate gel as inducing divalent and cations are used for wound healing, protein release and cell transplantation [20,21]. Due to the immense potential of microbial EPS in different sectors, they have many applications in the food, pharmaceutical and other industries. When compared to thermophiles, psychrophilic EPS has several benefits, including greater yield at short time and consistent emulsification [1,22]. Microbial polysaccharides are renewable, biodegradable and biocompatible. The relevant material properties make them attractive for various chemical, food, cosmetics and medical industries [23]. Microorganisms produce biopolymers such as polysaccharides, polyesters and polyamides. Most of them are hydrocolloids, and they are water-loving polymers that are easily dispersed in the water [24].

In the case of rhamnose-rich bacterial polysaccharides, they provide engaging biological activities that utilise their potential for a wide range of value-added applications for products such as cosmetics, pharmaceuticals, medical devices and functional foods [25]. Polysaccharides have physical and chemical properties such as water-binding capacity, high molecular weight, polyelectrolyte behaviour and in some cases, modulable molecular structural possibilities [26]. This enables them to exhibit various functional properties such as thickening, film formation, gelling, emulsion stabilisation, flocculation and production capability [27]. Furthermore, several studies have evaluated the non-toxicity of bacterial polysaccharides and their safety as cosmetic ingredients and are well documented [28,29].

Microbial polysaccharides find their applications in a wide range of non-food products and industrial purposes. In recent years, significant progress has been made in discovering and developing new bacterial polysaccharides with novel functional properties [30]. For example, microbial EPS promotes aggregation of soil particles, benefiting plants by keeping moisture in the environment and trapping nutrients [31]. EPS are hydrogenated polymers consisting of polysaccharides, proteins and DNA with unique properties. It possesses features such as biocompatibility, gelling and thickening ability for industrial applications [32]. Microbial polysaccharides are ionic, non-ionic and linear polysaccharides, to which side chains of various lengths and complexity are attached at regular intervals [33]. The same microbial chewing gums are produced by more than 1% of organisms [34,35].

World Health Organisation report estimated that worldwide around 50 million people have dementia, and there are nearly 10 million new cases every year [36]. The main symptoms are memory weakening and mental complaints, characterised by deposition of amyloid plaques in intra and extracellular neurofibrillary knots. Present treatment for AD has only modest benefits [37]. Hence, the improvement of drugs with significant effects has been of key importance. The current study aims to isolate and characterise an EPS produced by a soil-borne bacteria *B. amyloliquefaciens* RK3 strain isolated from the sugar industry effluent-contaminated soil, in order to assess its anti-AD potential.

## 2. Materials and Methods

### 2.1. Sample Collection and Isolation 

Sugar industry effluent-contaminated soil was collected from Bhodan (18°39′43.3″ N 77°54′35.2″ E), Nizamabad district, Telangana, India. The soil was collected aseptically at a depth of 5 cm and transported to the laboratory. The soil was dried in the oven at 30 °C to remove the moisture content. The dried soil was ground well and used for isolation. The soil was 10-fold diluted, plated on NA HiVegTM Agar (Nutrient Agar) plates and incubated at 37 °C/24 h. After incubation, the strain was selected and used for further studies. 

### 2.2. Biochemical and Molecular Identification of the Bacterial Strain

We employed conventional and molecular techniques to identify the bacterial strain. The biochemical characterisation was done by employing various physiological tests suggested in Bergey’s manual [38]. Molecular identification was performed by sequencing the 16S rRNA gene of the bacterial strain. Briefly, the genomic DNA was extracted by following the method of Palaniappan et al. [39], and the 16S rRNA gene was amplified using universal bacterial primers 27F and 1492R. BDT (v3.1) TM cycle sequencing kit was used to perform 16S rRNA amplicon sequencing on default parameters. Sequence analysis was performed on ABI 3730xl genetic analyser. The taxonomic relatives was identified by BLASTn search in the NCBI database and the ClustalW algorithm to align the relative sequences. Phylogenetic placement of the strain was confirmed by constructing the neighbour-joining tree in MEGA X [40].

### 2.3. Production Extraction and Purification of EPS

EPS production was carried out by following the recommended method of Sivasankar et al. [41] with some modifications. Briefly, the selected strain was inoculated in the basal medium containing (g L^−1^) casein (15 g), K_2_HPO_4_ (10 g), sucrose (20 g), yeast extract (5 g), sodium chloride (2.5 g), L-cysteine (0.5 g), MgSO_4_ 0.3 g; KH_2_PO_4_ 10 mg, pH 7.0 and Vitamin B1 as the added supplement for the enhanced microbial growth. The flasks were incubated in a rotary shaker (MaxQ 6000, ThermoFisher Scientific, Salem, India) at 28 ± 2 °C for three days. Viscosity and EPS production were monitored every 24 h. Upon incubation, the culture was centrifuged for 10 min at 12,000 rpm. The EPS was harvested by adding two volume of the cold-acetone to the cell-free culture supernatant and was kept overnight at 4 °C [42]. The precipitates were collected by centrifugation (12,000 rpm/4 °C/15 min) and used for further analysis. Subsequently, the collected precipitates were dissolved in Milli-Q water. The precipitate was added with an equal volume of cold-ethanol, and the precipitated EPS was collected by centrifugation, as mentioned above. The collected precipitates were pooled together, lyophilised and used for further analysis—the purification of EPS followed by Sun [42].

### 2.4. Characterisation of the EPS

#### 2.4.1. Estimation of Total Carbohydrates

The total carbohydrates present in the EPS samples were estimated using the phenol-sulfuric acid process [43]. Glucose was used as the standard.

#### 2.4.2. Thin-Layer Chromatography Analysis of EPS

The monosaccharide composition of the EPS was done after hydrolysis with Trifluoroacetic acid (TFA) [44]. First, EPS was hydrolysed with 2M TFA at 100 °C for two hours. Then, the released monosaccharides were analysed by TLC (MERCK Millipore, Germany). The solvent system was prepared by mixing ethyl acetate: acetic acid, 1-butanol and H_2_O in a ratio of 4:3:2:2 (*v*/*v*). The spots developed using spray reagent were prepared by adding 0.5% (*w*/*v*) 1-naphthyl ethylenediamine dihydrochloride in methanol with 5% sulphuric acid. The plate was dried at 120 °C for 10 min.

#### 2.4.3. UV-Visible Spectra, FT-IR and GC/MS Analysis of Purified EPS

The EPS sample was dissolved in Milli-Q water (5 mg in 2.5 mL *w*/*v*) and was used for UV-visible spectrophotometric analysis (THERMO Scientific Evolution 600). The sample was scanned between the wavelengths of 200 and 600 nm range. The lyophilised EPS was analysed with FT-IR to find out the functional groups present in the sample. In brief, the EPS sample (10 mg) was homogenised with potassium bromide at room temperature and was pelleted out by compression and analysed in FT-IR (Spectrum 100 Optica—PerkinElmer, Shelton, CT, USA) at the frequency range of 4000–400 cm^−1^ [45]. GC/MS analysis was performed to justify the monosaccharide composition of the EPS sample by following the standard method given by Centre for Cellular and Molecular Platform (C-CAMP), Bangalore, India. Initially, for monosaccharide composition, the EPS was treated with TFA (2 M) and was hydrolysed. The sample and sugar standards were reduced with KBH4 and again derivatised with MSTFA. Then, 1 µL of the sample was run on the GC/MS and the peaks were compared with that of the RTs of standards as well as mass spectral comparison in library. The linkage analysis was done by permethylating the sample with NaOH-DMSO and CH3I and was hydrolysed with TFA (2 M). The hydrolysed sample and standards of monosaccharides were reduced with NaBD4 to open up the sugar and derivatised with acetic anhydride and pyridine. Then 1 µL of the sample was run on the GC/MS and spectral peaks were identified based on masses and with the help of monosaccharide composition. Perkin-Elmer (Clarus S.Q. 8 GC/MS) with an autosampler instrument equipped with a capillary column RTX-5 30M, 0.32 mm ID was used for the analysis. 

#### 2.4.4. NMR and SEM Analysis

The nuclear magnetic resonance (NMR) analysis of purified EPS was performed using a 5 mm reverse probe Bruker AVANCE 400 MHz NMR spectrometer. The EPS was dissolved in deuterium oxide (D_2_O) at 10 mg mL^−1^ concentration and ^1^H NMR and ^13^C NMR spectra were measured. The NMR data were processed using Bruker TopSpin software (Waltham, MA, USA). In addition to that, scanning electron microscopy (SEM) and energy-dispersive X-ray spectroscopy (EDX) were also performed. The morphological aspect of EPS was studied through SEM. The EPS (1 mg mL^−1^) with aluminium stubs was dried in the air. The sample was sputtered using an SC7620 sputter coater. Field emission scanning electron microscopy (FE-SEM) coupled with EDX (TESCAN, VEGA 3 LMU instrument, Seoul, Korea) was used for this analysis.

### 2.5. Anti-Alzheimer Study of the EPS 

#### 2.5.1. Anti-Alzheimer Activity in Mice Models

The anti-Alzheimer activity of the EPS was assessed by following the recommended protocol [46,47]. In short, adult male albino mice (15–18 g) were purchased from the Madras medical college animal house, and work was carried out at Pharmacies college of Kakatiya University, Telangana state of India. All the animals were maintained under laboratory conditions and provided with the proper diet. The optimum temperature of the chamber was maintained at 25 ± 2 °C, while the humidity was maintained at 60 ± 5%. The Institutional Animal Ethics Committee approved the procedure and directions for carrying out this research (IAEC) (approval No. IAEC/53/UCPSC/KU/2018) and the animals were maintained according to the Committee’s regulations for control and supervision of experiments on animals (CPCSEA), Ministry of Environment and Forest, Government of India. The animals were housed in polypropylene cages for one week before starting the experiment, and every cage contained four or five mice. The animals were grouped into five groups, and each group contained 15 mice. Merely two lessons using mouse representations of AD have been evaluated by an exercise training effect. The experimental set-up was designed and executed as follows:

Group I: For three weeks, mice ingested 1 mL of saline via an oral path for Alzheimer’s activity control group.

Group II: Beta-amyloid solution was directly induced in the mice brain through stereotypic apparatus for three weeks (negative control).

Group III: Standard Rivastigmine was given (IP) (40 mg/kg) three weeks after beta-amyloid solution induction.

Group IV: Test solutions 200 mg/kg as given (IP) for three weeks after beta-amyloid solution induction. 

Group V: Test solutions 400 mg/kg as given (IP) for three weeks after beta-amyloid solution induction. 

Mice were fasted overnight and given anaesthesia to facilitate blood collection and the brain samples of mice. Subsequently, the brain tissue was quickly separated and further washed by isotonic saline solution. Weighing and homogenising the brain was done with 10% (*w*/*v*) ice cold 50 mM Tris-HCl and 300 mM sucrose-containing medium at pH 7.4. At 4 °C the samples were centrifuged at 2000 rpm for homogenisation. The supernatant was collected and stored at −80 °C for further use. Biochemical analyses were conducted to check the oxidative stress biomarker by nitric oxide concentration analysis, malondialdehyde (MDA) concentration, glutathione concentration and hydrogen peroxide concentration [48,49]. 

#### 2.5.2. Cognition and Behaviour Analysis of Mice Models 

The behavioural studies were also conducted on mice infected with disease and mice without symptoms as a negative control. The cognition and change in behaviour of different groups of mice were confirmed by various tests viz., jumping box test, rectangular maze test, y maze test study [50]. Histopathology of infected, controls and EPS-treated mice and acetylcholine esterase activity in the mice models were also performed [51]. 

## 3. Results and Discussion

### 3.1. Isolation and Identification of the EPS Producing Strain 

**Figure 1:**
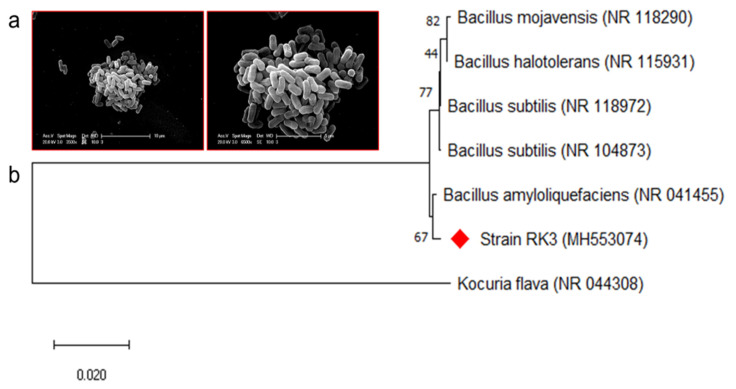
Representation of the morphology of *B. amyloliquefaciens* RK3 (**a**), and its phylogenetic placement with the closely related strains (**b**).

The strain RK3 was isolated from the soil samples collected from the area polluted by sugar factory effluents near Nizamabad, Telangana, India. The EPS production nature of the strain RK3 was initially confirmed on sucrose supplemented basal media [52,53]. Followed by the results, RK3 was selected for further studies. In addition, the strain RK3 was characterised by morphological tests. It was found to be a rod-shaped (Figure 1a) Gram-positive strain, growing well in 5% NaCl concentration and at 30 °C as the optimum growth temperature. Besides, the strain RK3 was found to be positive for the Voges-Proskauer test and citrate utilisation.

Interestingly the strain hydrolysed the starch very efficiently. Based on the morphological and biochemical test results, the strain RK3 was found to belong to the genus *Bacillus* [54]. Further, the strain was identified by using a molecular technique. The 16S rRNA gene sequence of the strain revealed that the strain RK3 has the closest similarity with the *Bacillus amyloliquefaciens*, and it was confirmed in the phylogenetic analysis (Figure 1b). The sequence was submitted to GenBank under the accession number MH553074. Based on the above justifications, the strain RK3 was identified as *B. amyloliquefaciens*. 

### 3.2. Production and Characterisation of the EPS

#### 3.2.1. Yield and Carbohydrate Content

Produced EPS was characterised for its chemical composition. The total yield of the EPS produced by *B. amyloliquefaciens* RK3 was 10.35 g L^−1^, better than EPS 7.75 g L^−1^ formed by *L. lactis* L2 [55]. The total carbohydrate content of the *B. amyloliquefaciens* RK3 EPS was 82.7%, and the higher carbohydrate content justifies the polysaccharide nature of the sample. 

#### 3.2.2. Estimation of Total Carbohydrates and Monosaccharide Composition 

**Figure 2:**
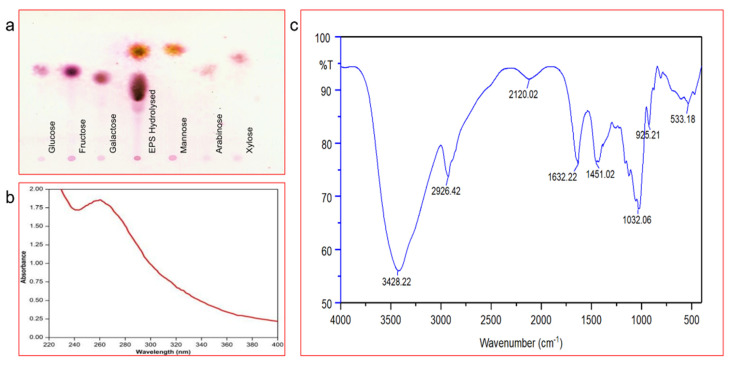
Shows the TLC analysis (**a**), UV-visible spectra (**b**) and the FT-IR analysis of the EPS produced by *B. amyloliquefaciens* RK3 (**c**).

The total sugar carbohydrates content was estimated and is shown in the Supplementary Material (see Figure S1). The monosaccharide composition of the EPS was analysed in TLC plate and the *B. amyloliquefaciens* RK3 EPS was found to contain mannose and galactose as the monosugar (Figure 2a). These results have been similar to previous studies from *B. amyloliquefaciens* LPL061 on EPS [56]. The results suggested that most of the carbohydrate was hydrolysed in the presence of TFA, due to its potential of hydrolysing glycosidic bonds without causing any drastic damage to monosaccharide units [57]. 

#### 3.2.3. UV-Visible Spectra and FT-IR Spectra

The UV spectrum of the EPS was recorded at the 200–800 nm absorption range, using methanol as blank. A strong absorption peak was obtained at 264 nm, representing the polysaccharide presence (Figure 2b). The absorption wavelength can correspond to –OH, –OCH3, –CO2, –COOH functional groups attached to an aromatic ring. The FT-IR spectrum of the EPS clearly shows peaks of carbonyl compounds. The peaks identified at 3428 cm^−1^ (–O–H), 1451 cm^−1^ (CH bending vibration) and 1032 cm^−1^ (C–O) can be related to the hydroxyl function group, like glucose or galactose, bending carbon functional group vibrations of alkanes and ether groups (Figure 2c). IR Spectrum at 2926.30 cm^−1^ and 1032.30 cm^−1^ represent aliphatic and C–O linkage that confirms the presence of polysaccharides [58,59]. The existence of phosphate, mannose, uronic acid, proteins, α or β as a whole, furanose or pyranose in EPSs can be preliminary to creating FT-IR spectra [60].

#### 3.2.4. GC/MS Spectra of Exopolysaccharide

**Figure 3:**
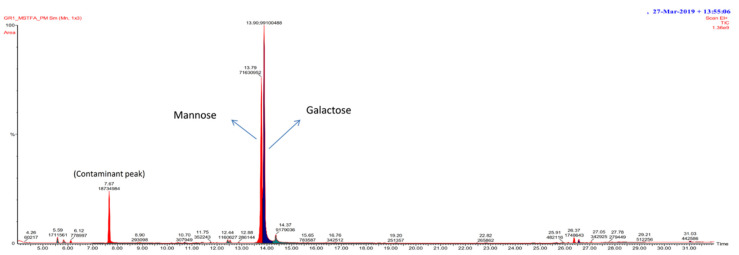
A GC/MS chromatogram displayed the monosaccharide composition of EPS polysaccharide.

GC/MS analysis was majorly used to decipher the attached monomeric units of polysaccharides [61]. GC/MS analysis possesses certain advantages to utilise it, such as rapid analysis with high selectivity, accuracy and fidelity with simple instrumentation [62]. The monosaccharide units present in the EPS were galactose with RT 12.79 and mannose with RT 12.88 (Figure 3). Similarly, in a previous study, mannose, sucrose, fructose and galactose were reported in the *B. circulans* EPS [54]. In another study, Liu et al. showed GC/MS trimethylsilylated EPS derivatives in a 33:1 molar ratio in the GC/MS assessment following hydrolysis of EPS by *B. licheniformis*. In addition, it has been abridged that polysaccharide can stimulate the anti-colon cancer effect with β-(1-6) linkages or lower Mw. In contrast, the arctic marine bacteria producing extracellular polysaccharides mainly comprises glucuronic acid, N-acetyl glucosamine, medium fructose, galactose, a small amount of rhamnose, glucose and mannose [63]. Chromatographic analysis has also shown that EPS from the RH-7 strain (marine bacterium *Rhodobacter*) is a heteropolysaccharide composed of galactose, glucose, glucuronic acid and rhamnose [64]. The monosaccharide hexopyranoside (galactosidase) and α-d-glucose were detected at RT 16. 28, 16.60, β-d-mannofuranose (RT 20.703, 23.41) and 43.101. β-GlcNac derivative (RT 14.890) and all the detected monosaccharides showed D-configuration [65].

##### Linkage Analysis of GC/MS

**Figure 4:**
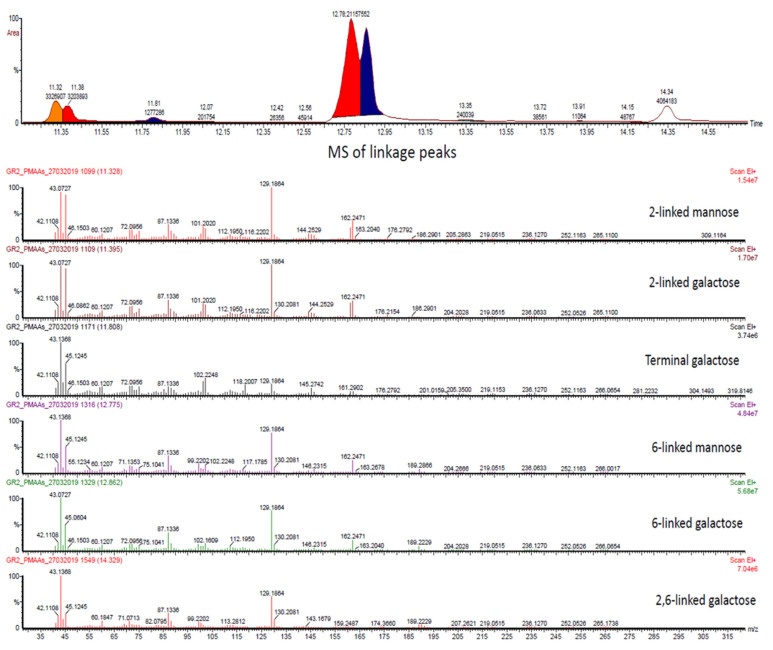
GC/MS chromatogram showing six different linkages (TOP) and corresponding MS spectra of linkages were presented (Bottom, at retention times 11.32, 11.39, 11.80, 12.75, 12.86 and 14.32 respectively). The linkages were determined according to the CCRC data base accessed on 27 march 2019 (https://www.ccrc.uga.edu/specdb/ms/pmaa/pframe.html#na).

The EPS are generally heteropolysaccharides, and their structural heterogeneity makes them resistant to various stress conditions [66]. Therefore, the identification through retention times of samples was compared with sugar standards, showing peaks for mannose and galactose. Further, linkage patterns were estimated based on mass spectra, monosaccharide compositions, and relative retention time. Results showed that EPS is a polysaccharide of majorly abundant residues as 2-links-Man, 2-links-Gal, T(terminal)-Gal, 6-links-Man, 6-links-Gal and 2,6-links-Gal (Figure 4 and Table 1).

Branch points and substitutions linkage and GC/MS results further provide the structural arrangement of EPS. The EPS was found to have 2- or 6-linked galactose with mannose branching at galactose residue. In another study, the monosaccharide composition from *B. amyloliquefaciens GSBa-1* EPS suggested that it might possess D-galactose, D-mannose. Additionally, their unique properties in structure influence the functional behaviour of microbe [67]. Similarly, the EPS of *L. plantarum* NTU 102 contained various monosaccharide units (arabinose, fructose, glucose, galactose, maltose and mannose) [68]. 

#### 3.2.5. ^1^H and ^13^C NMR Spectra Interpretation of EPS

**Figure 5:**
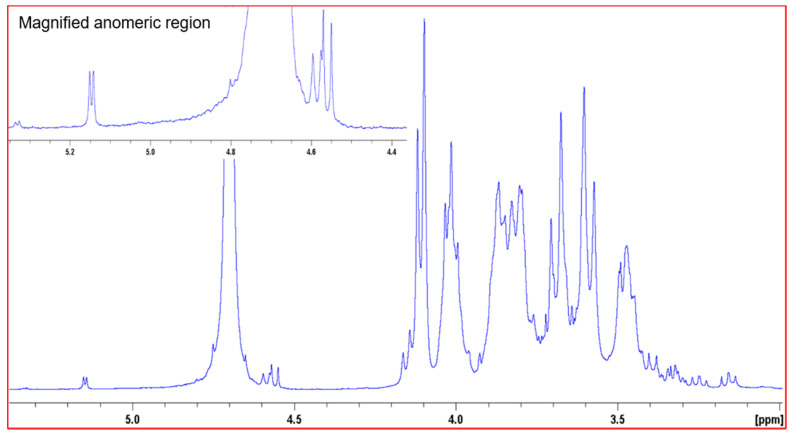
The expansion of ^1^H NMR spectra of EPS in the anomeric region (3.0–5.4 ppm), further expansion in the anomeric region (4.4–5.2 ppm) showed as the magnified portion.

The ^1^H NMR spectrum (Figure 5) displayed the characteristic anomeric (H1) signals in the anomeric region 4.0–5.4 ppm. In this anomeric region, a doublet was found at 4.55 ppm and another doublet was found at 4.58 ppm and these two peaks were identified β-d-Galactopyranose (β-d-Gal*p*), which should contain a linkage at its 6th/2nd position, and a Terminal (0) β-d-Gal*p* [69]. Since these peaks are slightly overlapped with the water signal, the intensities are incorrect and not shown in the figure (Magnified portion). Another doublet was found at 5.14 ppm identified as α-d-Mannopyranose (α-d-Man*p*) which contains the linkage at its 2nd and/or 6th position [70,71]. 

**Figure 6:**
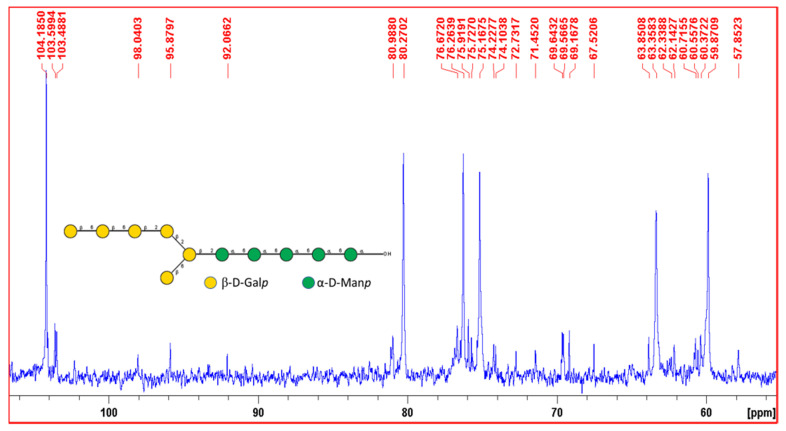
Representation of the expansion of ^13^C NMR spectra of EPS (50–110 ppm).

^13^C NMR spectra displayed NMR peaks in the range from 57.85 ppm to 104.18 ppm (Figure 6). The characteristic anomeric peak at 104.18 ppm was identified as anomeric C1 signal of β-d-Gal*p* which should be a terminal β-d-Gal*p* [72]. Another C1 anomeric peak at 103.59 ppm was identified as β-d-Gal*p* which should have a linkage at its 6th position and another peak at 103.48 ppm was also identified as β-d-Gal*p* [73,74]. Other anomeric peaks at 98.04 ppm, 95.8 ppm and 92.06 ppm were identified as α-d-Man*p* with the help of published literature and Carbohydrate Structure Database [75,76,77,78]. 

**Figure 7:**
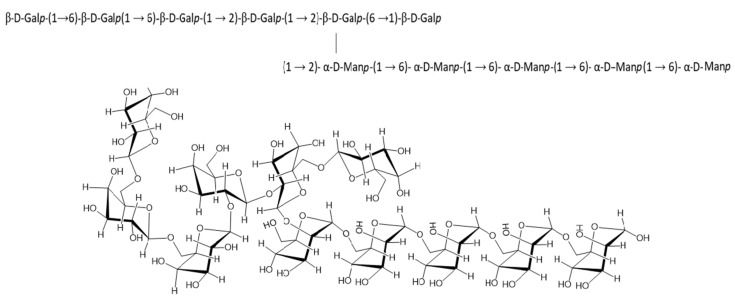
Representation of the predicted repetitive structural unit of EPS.

Based on the linkage analysis, monosaccharide composition analysis, NMR spectroscopic data, with the help of Carbohydrate Structure Database (CSDB) and simulations with the help of CSDB, the structure of the exopolysaccharide was elucidated and presented below (Figure 7) [78].

#### 3.2.6. SEM Analysis of the EPS

**Figure 8:**
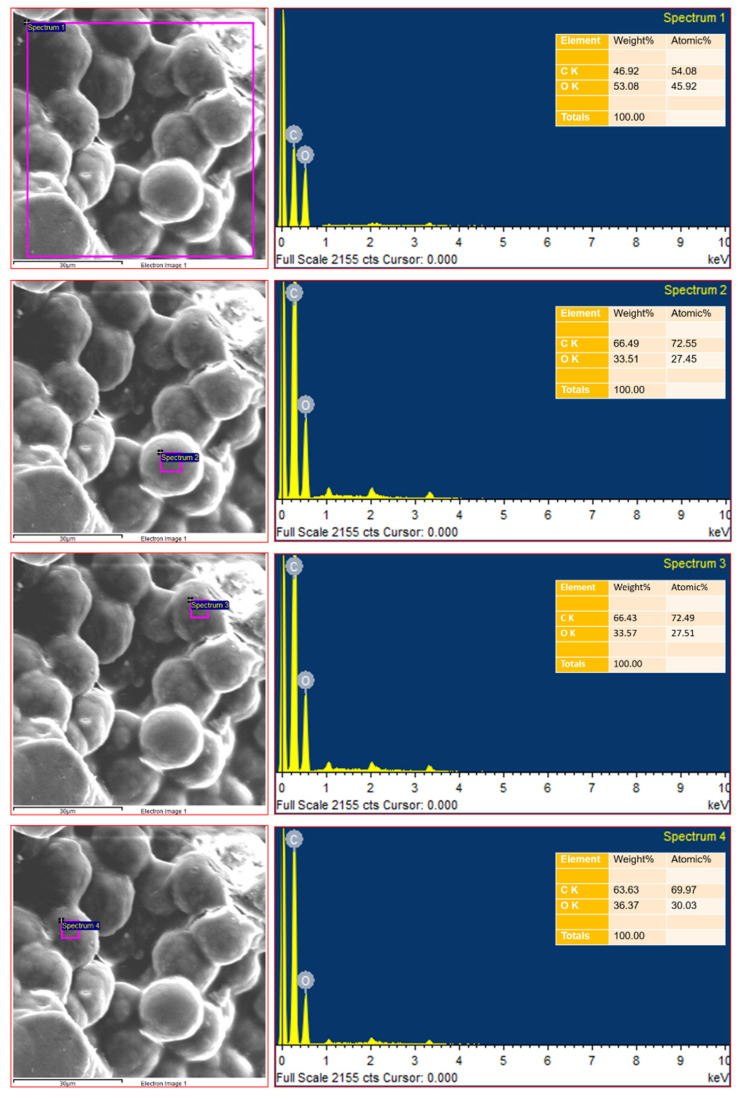
The morphology and the elemental composition of the EPS by SEM and EDX.

The morphological analysis of the EPS was done using SEM coupled with EDX. The SEM analysis investigated the morphological nature of EPS from *B. amyloliquefaciens* (Figure 8). The dense, porous and irregular design of EPS is discovered by the SEM study, used as a textured, thickening and stabilising agent to enhance water power and viscosity by the formulation of the matrix consistent with a hydrated polymer [79,80]. The porous nature of EPS has also been reported in the surface morphology and the elemental composition of EPS made from *S. thermophiles* [81]. EDX was carried out to study the elementary structure of EPS, showing different organic constituents in EPS, such as C and O (Figure 8). 

### 3.3. Anti-Alzheimer Study

#### 3.3.1. EPS Significantly Reduce the Convolutions of Alzheimer in Mice 

**Figure 9:**
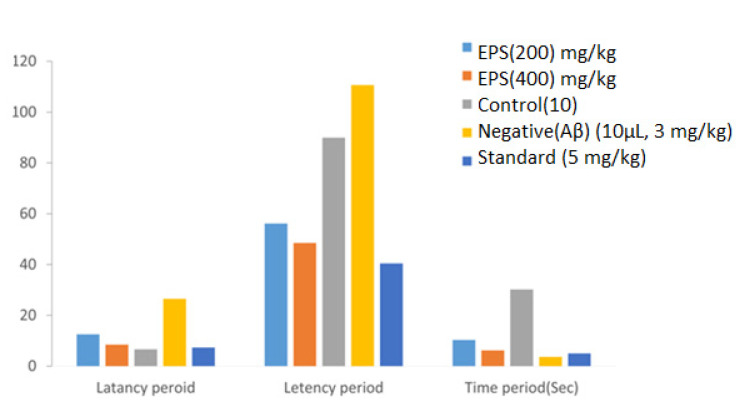
Representation of the latency period analysis in the mice for anti-Alzheimer’s activity (Latency period: *p* < 0.0001 when compared to the negative control group. ANOVA (one-way) followed by Bonferroni’s test).

AD is a neurodegenerative disease related to the loss of neurons, amnesia and reduced intellectual ability in affected individuals [82]. Many factors cause these diseases, especially oxidative stress, one of AD’s fugitive agents in various organisms [83]. The EPS showed a significant effect on cognition and aggregation of β-amyloid protein in mice brains with an effective dose of 200 mg/kg and 400 mg/kg (*w*/*w*) (Figure 9). Furthermore, after injecting the EPS directly into the mice, it significantly improved memory retention of learned mouse model tasks [68]. In the present study, the amnesic and antioxidant effect of EPS on mice was investigated at concentrations of 200 mg/kg and 400 mg/kg, showing EPS has a significant anti-amnesic effect. 

#### 3.3.2. Acetylcholine Esterase Activity

**Figure 10:**
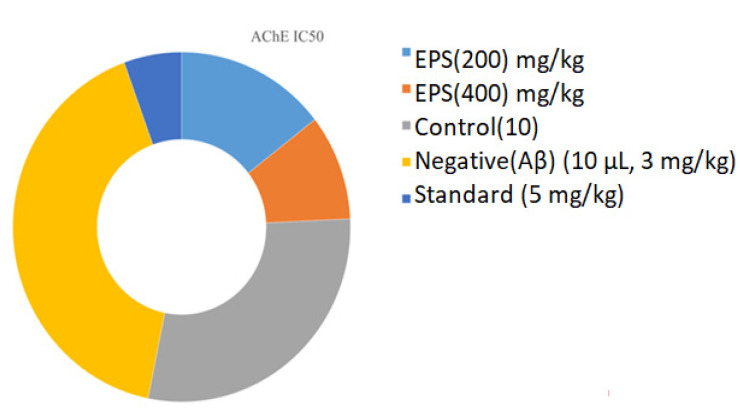
The acetylcholinesterase (AchE IC50) levels (Time period: *p* < 0.05, *p* < 0.001, *p* < 0.0001 when compared to negative control group. ANOVA (one-way) followed by Bonferroni’s test).

Biochemical analysis revealed that the acetylcholinesterase (AChE) levels are increased in the negative groups (0.97 ± 0.015 µg/mL) when compared to the control (0.68 ± 0.24 µg/mL) (Figure 10). Decreased AChE levels are observed in the EPS test sample and high inhibition was found in EPS 0.34 ± 0.01, 0.23 ± 0.04 µg/mL compared with respective samples. Nevertheless, there is a suggestion that AChE inhibitors may slow hippocampal atrophy, and disease progression may have disease-changing effects [84,85]. The AChE is the key enzyme responsible for the breakdown of the acetylcholine (ACh) in the normal brain. Inhibition of AChE is seen as a potential neurological condition treatment strategy for AD, ataxia, senile dementia, myasthenia gravis and Parkinson’s disease. Cognitive dysfunction together with AD treatment is used based on natural product donepezil and tacrine; these are synthetic medicines [86]. Researchers have reported that having adverse effects is associated with bioavailability and gastrointestinal disturbances, which are essential factors in finding best AChEIs from natural resources [87]. These findings revealed that the EPS of the *B. amyloliquefaciens* RK3 could be a better natural source advised for AD treatment. 

### 3.4. Effects of EPS on Behavior and Cognition of Mice Model

#### 3.4.1. Jumping Box Test

In the jumping box test, the effect of the EPS on the latency period of Alzheimer’s activity in mice was checked. There was an increase in the latency period in the negative control group, i.e., 26.51 ± 0.84 s, which was not treated with either EPS or any other effector molecule (Table 2). However, there is a reduction in the latency time in the population tested with EPS compared to a positive control (6.62 ± 0.37 s) and negative control. Thus, the jumping box test showed that the EPS reduced the Alzheimer latency period in mice. The decline in transfer latency time (TLT) indicates the memory-enhancing effect of drugs [88,89]. 

#### 3.4.2. Rectangular Maze and Y Maze Results

In the rectangular maze task, there was an increase in maze transverse period in the negative (110.6 ± 0.12 s) control group (89.91 ± 0.67 s) when compared to the control, and there is a decrease in a transverse period in groups treated with EPS (56.15 ± 0.32 s and 48.48 ± 1.25 s) (Table 3). Thus, our analysis compares to the maze test, and each trial represents a particular level of learning in mice [90].

In the Y maze test, there was an increase in maze transverse period in the negative (30.21 ± 0.14 s), control group (3.64 ± 0.24 s) when compared to control, and there is a decrease in a transverse period in groups treated with EPS (10.31 ± 0.24 s and 6.18 ± 0.35 s) (Table 3). The proportion of other references observed was estimated to be the ratio of factual to possible alternations [91]. 

### 3.5. Histopathology of Mice Brain 

**Figure 11:**
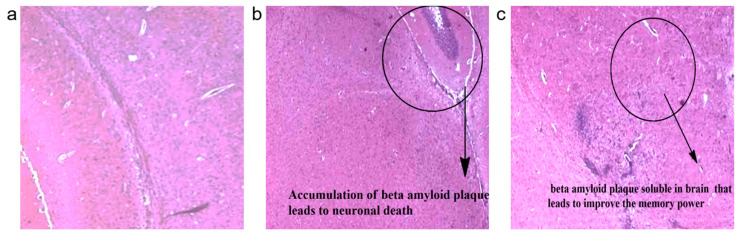
Representation of the histopathology of the mice brain samples (100× magnification). (**a**) Group-1 (control) received normal saline and did not expose to β-amyloid induction, and it appeared normal. (**b**) Group-2 received normal saline and did not expose to β-amyloid induction, and it has shown senile plaque formation (encircled portion showing the presence of accumulation of beta amyloid plaque). (**c**) Group-3 (Exopolysaccharide) received ethanolic fraction at a dose of 200 mg/kg orally and showed the plaque disappeared (encircled portion showing the absence of beta amyloid plaque).

After reperfusion for 24 h, mice were anaesthetised and killed by quick decapitation. The brains were separated and dipped in ice-cold saline solution for 10 min, kept overnight in fixation with 10% formalin at 28 °C, observed under a microscope at 100× magnification (Figure 11). The significant structures of memory development are the cerebral cortex and the hippocampus; the Hippocampal Aβ injection can result in a discrepancy in plasticity and synaptic transmission [92].

## 4. Conclusions

In this study, a novel EPS was isolated and characterised from the *B. amyloliquefaciens* RK3. The total yield of EPS after the purification was 10.35 g/L^−1^. The purified EPS showed medicinal applicability as anti-AD effects in mice models. A comparison of the treated and untreated mice indicated that EPS considerably decreased the amyloid level in the animals. The multiple repetitive mice model analysis shows that the EPS is an excellent anti-AD agent compared to some commercially available drugs. The anti-AD activity of the EPS might be a breakthrough in the treatment of other neurodegenerative disorders that occur due to oxidative stress. 

## Figures and Tables

**Table 1:** Linkage analysis of the EPS produced by the *B.* *amyloliquefaciens* RK3.

RT	Compound	Area	~Relative %
11.31	2-linked mannose	1,715,051	7.84
11.39	2-linked galactose	2,186,404	9.99
11.8	T-Galactose	2,327,302	10.64
12.79	6-linked mannose	8,432,888	38.54
12.88	6-linked galactose	5,788,062	26.45
14.3	2,6-linked galactose	1,430,488	6.54

**Table 2:** Representation of the latency period for control, negative, standard and test sample analysed by the jumping box test.

Samples	Group (mg/kg)	Mean ± SEM (s)
EPS	200	12.54 ± 0.60
EPS	400	08.48 ± 1.13
Control	10	6.62 ± 0.37
Negative	(Aβ) (10 μL, 3 mg/kg)	26.51 ± 0.84
Standard	(5 mg/kg)	7.3 ± 0.15

**Table 3:** Representation of the latency period and improvement in the spatial working memory for control, negative, standard and test samples.

Rectangular Maze Test		
Samples	Group (mg/kg)	Mean ± SEM (s)
EPS	200	56.15 ± 0.32
EPS	400	48.48 ± 1.25
Control	10	89.91 ± 0.67
Negative	(Aβ) (10 μL, 3 mg/kg)	110.6 ± 0.12
Standard	(5 mg/kg)	40.41 ± 0.83
**Y-Maze Test**		
**Samples**	**Group (mg/kg)**	**Mean ± SEM (s)**
EPS	200	10.31 ± 0.24
EPS	400	6.18 ± 0.35
Control	10	30.21 ± 0.14
Negative	(Aβ) (10 μL, 3 mg/kg)	3.64 ± 0.24
Standard	(5 mg/kg)	5.01 ± 0.31

## Data Availability

Not applicable.

## Supplementary Materials

The following are available online at https://www.mdpi.com/article/10.3390/polym13172842/s1, Figure S1: Represents the presence of total carbohydrates in the analysed EPS sample.
