# Supplementary figures and images for: Structural Characterisation and Assessment of the Novel Bacillus amyloliquefaciens RK3 Exopolysaccharide on the Improvement of Cognitive Function in Alzheimer’s Disease Mice
Source: Polymers (Basel). 2021 Aug 24;13(17):2842. doi: 10.3390/polym13172842 (PMC8434388; doi:10.3390/polym13172842)

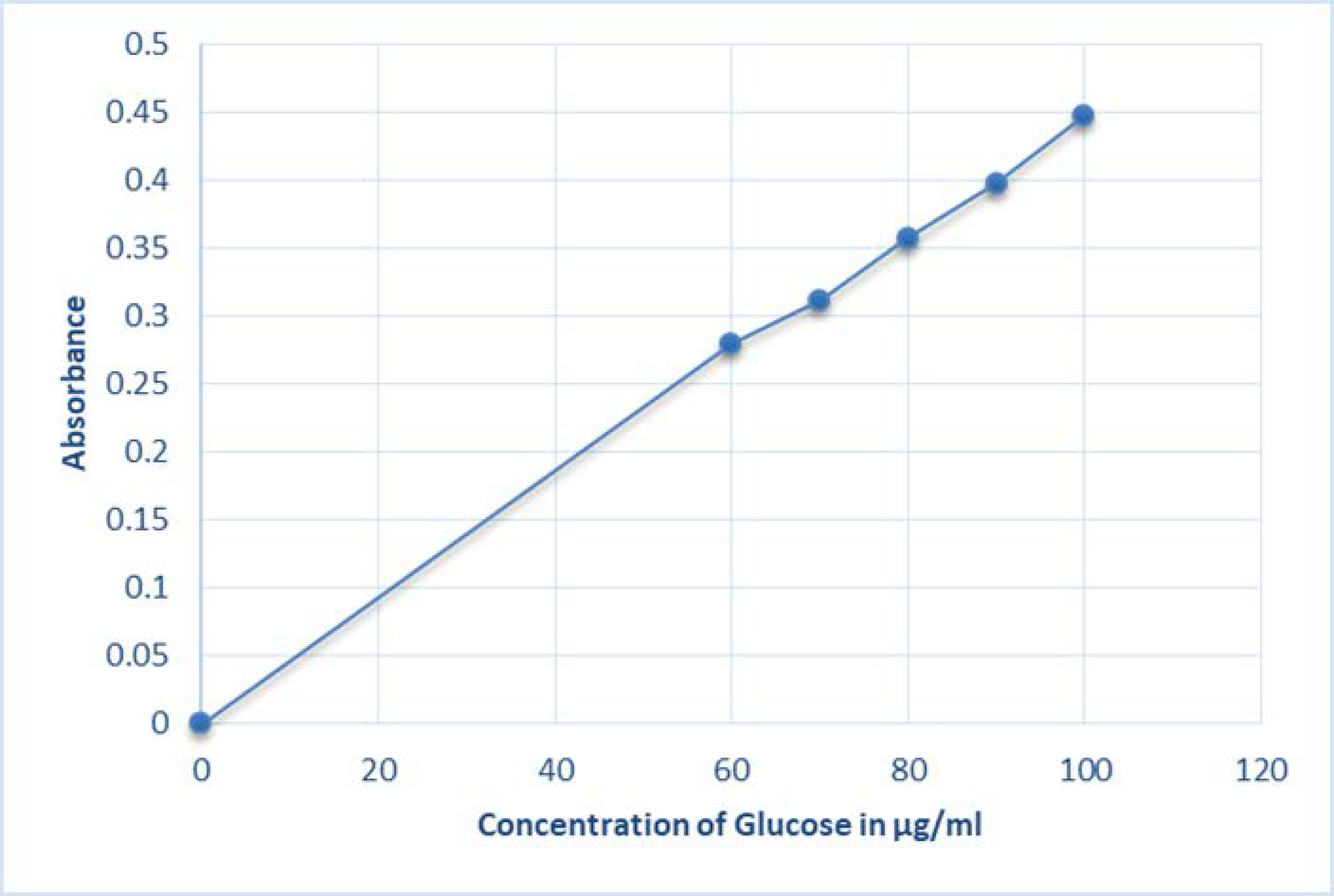

Supplement: Supplementary file 1 [file polymers-13-02842-s001.zip › polymers-1258350-supplementary.png]
